# An Emerging Role for Tubulin Isotypes in Modulating Cancer Biology and Chemotherapy Resistance

**DOI:** 10.3390/ijms18071434

**Published:** 2017-07-04

**Authors:** Amelia L. Parker, Wee Siang Teo, Joshua A. McCarroll, Maria Kavallaris

**Affiliations:** 1 Tumour Biology and Targeting, Children’s Cancer Institute, Lowy Cancer Research Centre, University of New South Wales, Sydney, NSW 2031, Australia; aparker@ccia.unsw.edu.au (A.L.P.); wteo@ccia.unsw.edu.au (W.S.T.); Jmccarroll@ccia.unsw.edu.au (J.A.M.); 2 Australian Centre for NanoMedicine, ARC Centre of Excellence in Convergent Bio-Nano Science and Technology, University of New South Wales, Sydney, NSW 2052, Australia

**Keywords:** tubulin, microtubule, cancer

## Abstract

Tubulin proteins, as components of the microtubule cytoskeleton perform critical cellular functions throughout all phases of the cell cycle. Altered tubulin isotype composition of microtubules is emerging as a feature of aggressive and treatment refractory cancers. Emerging evidence highlighting a role for tubulin isotypes in differentially influencing microtubule behaviour and broader functional networks within cells is illuminating a complex role for tubulin isotypes regulating cancer biology and chemotherapy resistance. This review focuses on the role of different tubulin isotypes in microtubule dynamics as well as in oncogenic changes that provide a survival or proliferative advantage to cancer cells within the tumour microenvironment and during metastatic processes. Consideration of the role of tubulin isotypes beyond their structural function will be essential to improving the current clinical use of tubulin-targeted chemotherapy agents and informing the development of more effective cancer therapies.

## 1. Introduction

The microtubule cytoskeleton is an extensive network of filaments that span the cell interior. Microtubules are composed of αβ-tubulin heterodimers that associate to form protofilaments, which then laterally associate to form microtubules [1]. These highly dynamic structures are constantly lengthening and shortening throughout all phases of the cell cycle by the addition and removal of tubulin heterodimers at the microtubule ends. The manner in which tubulin heterodimers are orientated in microtubules gives rise to a polar molecule that differs in its structure and kinetics at each end of the microtubule [1]. The dynamics of tubulin heterodimer addition and release are slower at the minus end of the microtubule, which terminates in α-tubulin proteins, compared with the plus end of the microtubule, which terminates with β-tubulin proteins capped in the GTP-bound state [2,3]. Therefore, microtubules are nucleated at their minus ends while their plus ends more dynamically lengthen and shorten [1]. The transition of a microtubule from a period of lengthening to one of shortening is termed catastrophe, while the restoration of microtubule lengthening following a period of shortening is termed rescue. Any single microtubule will dynamically transition between any of these phases over its lifetime. In addition, interactions between microtubules and an extensive network of proteins regulate their stability and dynamicity [4]. Readers are referred to several extensive reviews of microtubule dynamics and its regulation for more detailed description of these processes [5,6].

During interphase, the majority of microtubules are nucleated at the centrosome and radiate towards the cell periphery [3]. One of the major functions of interphase microtubules is their involvement in maintenance of cell shape and trafficking of proteins and organelles [4]. Motor proteins utilize the microtubule cytoskeleton for translocating cell components on microtubule tracks, and co-ordination of this process occurs by protein–protein interactions with other adaptor proteins [4].

During mitosis, the microtubule network remodels to form the mitotic spindle, and the dynamic nature of the microtubule filaments enables correct segregation of the chromosomes. Failure to correctly attach or segregate the chromosomes initiates cell cycle arrest at the mitotic checkpoint, and can lead to the initiation of apoptosis [7]. The tubulin-binding agent (TBA) class of chemotherapeutics, which include taxanes, *vinca* alkaloids and epothilones, directly target β-tubulins to suppress the dynamics of spindle microtubules, thereby disrupting chromosomal segregation to induce cell cycle arrest and cell death [8]. Particular classes of tubulin binding agents have also more rarely been detected binding to both α- and β-tubulin proteins at the interface between two dimers [9].

In humans, microtubules are composed of mixed combinations of eight α-tubulin isotypes and seven β-tubulin isotypes [10]. The different tubulin isotypes are encoded by different genes on distinct chromosomes and possess specific tissue and developmental distributions [11,12]. Tubulin isotypes share a high degree of structural homology but are distinguished from one another by highly divergent sequences at their carboxy-terminal (C-terminal) tail [13]. The N-terminal and intermediate domains of the protein form a highly conserved and rigid globular tubulin body that stack to form protofilaments, while the C-terminal tail of the protein is a highly disordered peptide 18–24 amino acids in length. These C-terminal tails are sites for post-translational modifications and mediate interactions with a range of proteins [4], which collectively impart unique functionality to each tubulin isotype [4]. A co-ordinated cohort of chaperones and post-chaperonin cofactors assists the folding of nascent peptides into the highly rigid globular fold adopted by the tubulin body while also controlling the formation of tubulin heterodimers [14,15]. Microtubules do not readily accommodate deviations in the tubulin fold and therefore obtaining correct tubulin fold is critical in regulating microtubule structure and dynamics. Furthermore, the associations of tubulins with their post-chaperonin cofactors also regulates the dynamic exchange of tubulin heterodimers between the polymerised and soluble tubulin pools and enables this process to operate far from equilibrium [14]. However, the tubulin globular fold is highly conserved and how tubulin folding might contribute to regulating tubulin isotype composition is largely unknown.

In a range of cancers with different primary sites, the tubulin isotype composition of cancer cells is perturbed compared with the surrounding healthy tissue. This aberrant expression involves increased or decreased expression of one or more tubulin isotypes, as well as the expression of tubulin isotypes that are not normally detectable in the healthy tissue [16]. Importantly, this aberrant expression of different tubulin isotypes correlates with patient outcome and treatment response. This review focuses on the current knowledge of the role of tubulin isotypes in chemotherapy drug resistance, disease aggressiveness and tumour biology. The majority of studies in this field have focussed on the importance of β-tubulin proteins, largely due to the specificity of tubulin-binding agents for β-tubulin proteins together with a dearth of tools to interrogate the importance of α-tubulin isotypes in this context. However, undoubtedly, α-tubulin isotypes, similar to β-tubulin isotypes, will also be increasingly associated with cancer patient outcome with further advances in our understanding of tubulin and tumour biology.

## 2. Tubulin Isotype Expression in Cancer

Altered expression of tubulin isotypes has been observed in a range of cancers. Analysis of clinical specimens has shown that high expression of several tubulin isotypes, namely βI-, βII-, βIII-, βIVa-, and βV-tubulin, correlates with aggressive clinical behaviour, chemotherapy drug resistance and poor patient outcome in many cancers (Table 1). Although tubulin protein levels are predominantly regulated post-translationally such that protein levels of tubulin isotypes poorly reflect the transcript levels [16], changes in the tubulin isotype expression have been characterised at both the transcript [16,17,18] and protein level [16,19,20,21,22,23,24,25,26,27,28].

Aberrant βIII-tubulin expression has been the most comprehensively characterised of the tubulin isotypes in this context and since the first report of high expression levels of βIII-tubulin in paclitaxel-resistant ovarian cancer [29], growing clinical evidence indicates that βIII-tubulin expression is involved in resistance to taxanes and *vinca* alkaloids in a range of tumour types, including lung, breast, ovarian and gastric cancers (Table 1). While the majority of these studies have identified that high expression of this isotype correlates with poor patient outcome, the opposite effect has been noted in ovarian clear cell adenocarcinoma (Table 1). This contrasting prognostic and predictive value of βIII-tubulin expression in taxane response in ovarian cancer subtypes suggests a tumour-type specificity that is incompletely understood. Multiple studies have shown that the expression of βIII-tubulin correlates with poor response to taxane-based chemotherapy and a correlation of βIII-tubulin positivity with more advanced clinical stage of ovarian cancer [19,20,21,28,29,30,31,32,33]. In contrast, in ovarian clear cell adenocarcinoma, overexpression of βIII-tubulin predicts a good response to taxane-based chemotherapy and, among patients treated with taxane-based chemotherapy, cases with higher βIII-tubulin expression were associated with a significantly more favourable prognosis compared with those having lower βIII-tubulin expression [34]. These contrasting findings reveal the complexity by which altered βIII-tubulin expression may influence TBA responsiveness, and highlight the need to understand the factors determining the prognostic and predictive value of tubulin isotype expression in TBA efficacy.

Altered expression of βI-, βII-, βIVa-and βV-tubulins has also been associated with resistance to TBAs in a number of cancer types (Table 1). However, to date, clinical studies investigating these specific tubulin isotypes in cancer have been limited and further investigation is required to define their contribution to drug resistance phenotypes.

While mutations in the β-tubulin isotypes are not clinically prevalent [35], a recent study in breast cancer patients identified several mutations in βI-, βIIa- and βIVb-tubulin isotypes that modify the isotype sequence to resemble that of βIII-tubulin, and the authors propose that these structural permutations are likely to impact clinical outcome in a similar manner to altered βIII-tubulin expression [36].

β-Tubulin isotype expression in tumour-associated stroma has not been extensively studied. One recent study identified that treatment of ovarian cancer with neoadjuvant chemotherapy primarily reduced the βIII-tubulin expression in stroma, and that a reduction in βIII-tubulin expression levels in both the tumoural and stromal compartment were each associated with increased survival [28]. How changes in the tubulin isotype composition of stroma may influence disease progression and drug response warrants further investigation.

### 2.1. Tubulin Isotypes and Drug Resistance

Clinical observations that altered expression of β-tubulin isotypes in cancer are associated with altered sensitivity to TBAs are supported by in vitro studies noting altered expression of βII-, βIVa-, βIVb and βV-tubulin isotype expression in a range of drug-resistant cancer cell lines [29,40,49,50,51,52,53,54,55,56,57,58,59]. Forced genetics approaches further substantiate these findings and have illustrated that β-tubulin isotypes differentially affect the efficacy of TBAs in a cell type-dependent manner [56]. βII-Tubulin and βIII-tubulin isotypes confer resistance to taxanes in non-small cell lung cancer (NSCLC) cells, while suppression of βII-tubulin or βIVb-tubulin sensitises NSCLC [49,50] and pancreatic cancer cells [60] to *vinca* alkaloids. Conversely, suppression of βII-tubulin specifically sensitises NSCLC cells to epothilone B [49,50]. These findings conflict with other studies showing that βIII-tubulin but not βI-tubulin or βII-tubulin expression affected epothilone sensitivity in NSCLC and breast cancer cell lines [61]. Conversely in ovarian cancer cells, suppression of βII-tubulin does not sensitise cells to taxanes or *vinca* alkaloids but does sensitise them to the non-taxoid microtubule stabilising agents laulimalide and peroluside [56]. Further delineation of the factors that determine the specificity of the tubulin isotypes for influencing TBA responses across different cell types is required.

Compared with other β-tubulin isotypes, the βIII-tubulin isotype has been the most extensively studied tubulin isotype for its role in drug resistance, and growing clinical (Table 1) and in vitro evidence supports its role in mediating broad-spectrum drug resistance. In vitro studies have identified a role for this isotype in mediating resistance to DNA damaging agents, antimetabolites and topoisomerase inhibitors in NSCLC cells, melanoma and pancreatic cancer cells in addition to influencing taxane and *vinca* alkaloids resistance [49,62,63,64,65,66], suggesting that this isotype plays a broader role in chemotherapy resistance mechanisms that extend beyond its function as a binding target for TBAs. This is supported by observations that βIII-tubulin expression levels do not equally modulate the sensitivity of cancer cells to all TBA subclasses [56].

Other studies overexpressing β-tubulin isotypes have yielded conflicting results. Overexpression of βIII- or βV-tubulin in CHO cells increases the resistance of these cells to paclitaxel [52,67] while other studies indicate that overexpression of βI-, βII-, and βIVb-tubulin does not affect TBA resistance in these cells [68]. In addition, overexpression of βIII-tubulin failed to confer resistance to TBAs in prostate cancer [69]. These discrepancies may reflect the complex autoregulation of β-tubulin isotype expression [70], their collective and combinatorial influence on drug sensitivity [71], and the importance of treatment regime and disease stage in the clinical observations associated with altered β-tubulin isotype expression [72]. While forced genetics approaches have yielded important insight into the contribution of β-tubulin isotypes in drug resistance, more advanced understanding of the mechanisms that drive altered isotype expression and tools to manipulate these endogenous regulatory processes in cancer cells would make valuable contributions to the field.

### 2.2. Microtubule Dynamics and Chemotherapy Resistance

The most characterised mechanisms of action of TBAs are their drug-induced suppression of microtubule dynamics, the induction of cell cycle arrest and the induction of cell death [8]. The different dynamic properties of the β-tubulin isotypes may alter the sensitivity of microtubules to these agents and thereby explain how altered β-tubulin isotype composition impacts drug responsiveness in the clinical and preclinical setting. In cell-free studies using isolated tubulin, microtubules composed of the βIII-tubulin isotype are more dynamic than microtubules composed of βII or βIV-tubulin isotypes, which are associated with more stable microtubules [73,74,75]. In this manner, the presence of βIII-tubulin may support microtubule dynamics even in the presence of TBAs, thus contributing to resistance to the primary mechanism of action of this drug class. More recent studies indicate that tubulin isotypes differ principally in their catastrophe frequency [76,77,78]. For example, microtubules composed of βIII-tubulin have a 1.5–3 fold higher catastrophe frequency compared with those composed of βII-tubulin [78]. In this respect, altered expression of β-tubulin isotypes may fine-tune the catastrophe frequency of the microtubule, thereby altering basal microtubule dynamics and the impact of chemotherapeutics that suppress microtubule dynamics. Microtubule-associated proteins can further influence microtubule dynamics in a tubulin isotype-specific manner. In the presence of microtubule-associated proteins, microtubule assembly is inhibited and proceeds faster in the presence of βIII-tubulin compared with other isotypes [79,80,81]. Therefore, the collective influence of the tubulin isotype composition and the microtubule associated proteins that spatiotemporally interact with the microtubule network may regulate basal microtubule dynamics and the response of the microtubule network to TBAs in cancer cells.

This complexity in the regulation of microtubule dynamics may explain why observations that the β-tubulin isotype composition intrinsically modulates microtubule dynamics in cell-free systems have not been unequivocally supported by observations within the more complex intracellular environment. In the absence of tubulin-binding agents (TBAs), depletion of βIII-tubulin does and does not influence interphase microtubule dynamics in mammary epithelium and lung cancer cells, respectively, while βII-tubulin levels do not affect microtubule dynamics in the latter cell type [82,83]. However, in the presence of TBAs, suppression of βIII-tubulin expression consistently reduces the efficacy of TBAs (ixabepilone, eribulin, paclitaxel, and vincristine) in suppressing microtubule dynamics in a number of cell types, concurring with observations in cell-free systems [74,82,84,85,86,87,88]. Conversely, observations that the βIII-tubulin expression level alters the sensitivity of microtubules to the destabilising effects of colchicine [89,90] could not be recapitulated in breast cancer cell lines [88]. While microtubules composed of βIV-tubulin are also more resistant to paclitaxel-mediated microtubule stabilisation and α-βII-tubulin microtubules are most sensitive to vinblastine in the presence of MAPs in cell-free systems compared with other tubulin isotypes [79,80], these findings have not been confirmed in the more complex intracellular environment.

Differences in the TBA-binding properties of different tubulin isotypes may partially explain how altered isotype composition may give rise to a drug resistance phenotype. Microtubules composed of mixed tubulin isotypes or purified βII-tubulin proteins bind fewer taxol molecules compared with βIII-tubulin or βIV-tubulin [74]. Conversely, the microtubule destabilising agents vincristine, vinorelbine and nocodazole interact more weakly with βIII-tubulin than with other β-tubulin isotypes in vitro, while nocodazole, similar to colchicine, binds most strongly to α-βIV-tubulin [81,91]. Estramustine [92] and eribulin [85] also bind to microtubules composed of α-βIII-tubulin less efficiently than other β-tubulin isotypes [92]. Therefore, altered tubulin isotype expression in cancer cells may directly contribute to the sensitivity of the microtubule cytoskeleton to tubulin-binding chemotherapy agents to subsequently impact treatment efficacy.

Other classes of chemotherapy agents have also been observed to directly interact with tubulin proteins. Although DNA-damaging agents are not canonical tubulin binding agents, cisplatin is capable of binding to tubulin through the protein’s GTP site [93] and inhibit microtubule assembly in cell free systems [94]. Whether this effect explains the role for βIII-tubulin in conferring resistance to DNA-damaging agents [61,63,66,95] has not been directly examined.

Conformational changes induced upon drug binding and differences in the residues that stabilise drug binding are believed to account for the impact of different tubulin isotype compositions on the dynamic and drug sensitivity behaviours of microtubules [77,90,96,97,98]. Recent evidence indicates that the unique dynamic characteristics of the individual isotypes are encoded within the tubulin body, despite their high degree of structural homology in this region of the tubulin protein [78]. The intermediate domain of the tubulin body has been proposed to regulate microtubule dynamics through its conformational state and by coupling the tubulin conformation to the tubulin GTP hydrolysis cycle [99,100]. In addition, the isotype defining region of the tubulin proteins, the C-terminal tail, which regulates microtubule dynamics through interactions with the intermediate domains of neighbouring tubulin isotypes to destabilise microtubules [101,102,103,104,105,106,107,108,109,110,111,112,113,114,115] may also contribute to the dynamic features of microtubules through differences in the conformational space covered by these flexible regions of the protein and by interacting with neighbouring tubulin heterodimers in an isotype-specific manner [101,102,103,116]. While the disordered nature of the tubulin C-terminal tail has hindered direct experimental characterisation of tail-body interactions proposed by these molecular dynamics simulations, evidence that the C-terminal tail region differentially affects microtubule assembly between isotypes [117] supports the notion that this region of the protein may significantly contribute to isotype-specific microtubule dynamics. Similarly, post-translational modifications and isotype-specific interaction with microtubule associated protein interactions in this C-terminal tail region may also confer different dynamic properties to the tubulin isotypes [118]. Furthermore, the ability of tubulin-binding agents to preferentially relocalise subsets of plus-end tracking proteins [119] may amplify the influence of particular tubulin isotypes on tubulin-binding agent activity.

Overall, differences in the sensitivity of different tubulin isotypes on the ability of TBAs to suppress microtubule dynamics may partially explain the associations between altered isotype composition and resistance to these agents.

### 2.3. Tubulin Isotypes in Tumour Biology

While it is clear from the studies described in the preceding section that the β-tubulin isotype composition affects microtubule dynamics and the sensitivity of microtubules to TBAs, this simplistic model of drug resistance fails to explain how an individual β-tubulin isotype, βIII-tubulin might confer resistance to diverse non-tubulin targeting agents such as DNA-damaging agents, antimetabolites and topoisomerase inhibitors [61,63,66,95]. In addition, βIII-tubulin expression regulates the tumourigenic and metastatic potential of NSCLC and pancreatic cells in vivo [31,63,66,120], further supporting a broader role for this β-tubulin isotype in tumour biology. The importance of βIII-tubulin in the progression of the primary tumour appears to be cancer specific, since suppression of βIII-tubulin did not affect primary tumour growth in a breast cancer model [121].

Intracellular processes that enable tumour progression also facilitate drug resistance, thereby providing a common foundation from which altered expression of β-tubulin isotypes may impact both drug resistance and disease aggressiveness. Central to these are oncogenic, stress response and cell death signalling processes that enable cancer cells to survive and proliferate within the harsh tumour microenvironment and in response to drug treatment. Involvement of β-tubulin isotypes in the signalling processes that regulate the progression of the primary tumour are discussed in more detail below.

## 3. Tubulin and Oncogenic Signalling

Although tubulin isotypes are aberrantly expressed in many types of cancers, there is no evidence to support their classification as an oncogene, which by definition is a gene that when genetically altered, enables it to drive the transformation of normal cells. Nevertheless, several studies have shown that βIII-tubulin may serve as downstream targets of several known oncogenic pathways. βIII-Tubulin is induced by mutant Ki-ras2 Kirsten rat sarcoma viral oncogene homolog (KRAS) expression and also in response to Ki-ras2 Kirsten rat sarcoma viral oncogene homolog (EGFR) stimulation by a post-translational mechanism [24]. Correlations between high βIII-tubulin expression and KRAS mutant tumours in NSCLC [24] suggests that βIII-tubulin may potentially contribute to Ras-induced transformation, although this hypothesis remains to be investigated in vivo.

βIII-Tubulin expression has also been associated with the phosphatase and tensin homolog (PTEN) signalling pathway. In prostate cancer, *PTEN* deletions correlate with elevated βIII-tubulin expression in prostate cancer, suggesting that altered βIII-tubulin expression may result from PTEN-mediated reprogramming events in tumour initiation [48]. The same study also showed that βIII-tubulin expression is strongly associated with ERG expression and *TMPRSS2:ERG* rearrangement in prostate cancer [48]. In colon cancer, Xiao and colleagues reported that inhibiting the Protein kinase B (AKT) or the extracellular-signal-regulated kinase (ERK) signalling pathways downregulates expression of βIII-tubulin, indicating that βIII-tubulin could be regulated by both AKT and ERK pathways [122]. Suppression of βIII-tubulin expression in NSCLC cells has been demonstrated to increase PTEN expression, which acts upstream of AKT to inhibit its phosphorylation (activation) [123]. Functionally, this correlated with increased sensitivity to anoikis (a form of programmed cell death) and reduced tumour growth in mice [123]. The PTEN/phosphoinositide 3-kinase (PI3K)/AKT signalling axis is commonly dysregulated in tumour cells and is strongly implicated in tumourigenesis and metastasis to promote cancer cell survival and proliferation during tumour progression and in response to chemotherapy agents. Further delineation of how βIII-tubulin may modulate the activity of this important signalling pathway is required.

Overall, these observations raise the notion that tubulin isotypes may act as permissive mediators of oncogenic signalling to enable tumour progression. The context in which they contribute to oncogenic signalling and the impact of their involvement remains to be comprehensively characterised for different disease settings.

## 4. Tubulin and Microenvironmental Stress Response

### 4.1. Tubulin and Hypoxia

Dramatic microtubule remodelling occurs in hypoxic conditions, suggesting an important role for the microtubule network in hypoxic adaptation [124,125,126]. While the importance of specific tubulin isotypes in this context remain to be fully defined, emerging evidence supports a specific role for βIII-tubulin in hypoxic adaptation. In particular, hypoxia-inducible factor 1-α (HIF1α) binds to an E-box motif located within the 3′UTR of *TUBB3* and induces its expression in ovarian cancer cells [127]. Similarly, HIF1 and HIF2a interactions with overlapping HIF response elements within the *TUBB3* 3’UTR suppress and induce *TUBB3* expression in glioblastoma in normoxia and hypoxia, respectively [128]. Hypoxic upregulation of βIII-tubulin expression in ovarian cancer cells is linked to increased HIF2α and SOX9 transcriptional activity [129]. However, these effects are cell type specific, depend upon the epigenetic status of the *TUBB3* 3′UTR and are also influenced by the basal βIII-tubulin expression level [55,127]. The expression of βIII-tubulin is also regulated by the interaction of the *TUBB3* transcript with the RNA binding protein Hu antigen (HuR) [130], which is involved in the recruitment of mRNA transcripts to polysomes and has been demonstrated to be involved in HIF1α stabilization [131]. Necrotic tumour regions have been shown to express high levels of βIII-tubulin, suggesting a functional role of βIII-tubulin in cellular adaptation to hypoxia [132]. Whether altered expression of βIII-tubulin in response to hypoxia has functional consequences in promoting hypoxic adaptation is unknown.

### 4.2. Oxidative Stress and Microtubules

Oxidative stress depolymerises microtubules and reduces the microtubule assembly rate in cardiomyocytes and neurons [133,134] suggesting that the microtubule cytoskeleton may play an important role in restoring redox balance. However, whether the microtubule network mediates redox homeostasis in cancer cells and in response to oxidative-stress inducing agents has not yet been demonstrated.

Comparative analysis of the structure of the different tubulin isotypes suggests that individual isotypes may contribute to cellular sensitivity to oxidative stress by acting as redox switches [135]. These have been specifically identified in βIII-, βV- and βVI-tubulins wherein Ser/Ala124 has been substituted for cysteine, and Cys239 substitutes serine. These individual isotypes have been specifically identified as potential sensors of oxidative stress [135]. Cys239 is readily oxidised and its oxidation inhibits microtubule assembly and stability [135].

Hence, changes in tubulin isotype composition may influence microtubule stability in response to oxidative stress to support microtubule integrity and cell survival under a harsh tumour microenvironment.

Oxidative stress can be induced by the harsh tumour microenvironment, high metabolic activity of tumour cells and by radiation and chemotherapeutic treatments. DNA damaging agents are particularly reliant on the induction of oxidative stress for their efficacy [136,137]. βIII-Tubulin and the DNA damage repair enzyme excision repair cross-complementation group-1 (ERCC1) co-operatively influence patient response to taxane combination therapy [41], however the mechanisms underlying this co-operative effect are unknown. Direct interactions between βIII-tubulin and mediators of the oxidative stress response proteins glutathione S-transferase µ4 and dimethylaniline monooxygenase 4 have been reported in ovarian cancer cells [138]. In neurons, suppression of βIII-tubulin expression sensitises cells to reactive oxygen species (ROS)-induced neurotoxicity in an isotype-specific manner, suggesting that it may protect cells from oxidative stress [139]. Therefore, altered β-tubulin isotype expression in cancer may protect cells from oxidative stress to confer chemotherapy resistance and enable tumour progression.

### 4.3. Tubulin Isotypes and Metabolism

Microtubules have been proposed to act as sensors of the energy state of the cell [140,141], however the underlying mechanisms and involvement of specific tubulin isotypes is unclear. Recent studies have demonstrated that tubulin is capable of interacting with, and blocking the voltage dependent anion channel (VDAC) within the mitochondrial outer membrane, thereby regulating ATP and metabolite compartmentalisation to inhibit oxidative phosphorylation and promote glycolysis [142,143,144,145,146]. Molecular dynamics simulations have revealed that an initial interaction between the tubulin body and VDAC is followed by insertion of the tubulin C-terminal tail within the channel lumen to regulate VDAC selectivity and voltage sensitivity [142]. The different tubulin C-terminal tail sequences together with their associated post-translational modifications are likely to regulate the channel permeability and selectivity [142,145,147]. VDAC is a component of the mitochondrial interactosome and regulates the mitochondrial membrane potential [148] to influence the committal stages of apoptotic cell death [149] and the overall potential for cells to proceed towards cell death in response to stimuli.

The general ability of tubulin proteins to bind to a range of glycolytic enzymes and regulate their activity as well as to reciprocally influence microtubule dynamics has been well established in cell-free systems [150,151,152,153,154,155,156,157,158,159,160] and in ovarian cancer cells [138] but clarity on the influence of specific isotypes and the functional implications of these interactions has been lacking. A more recent study in NSCLC cells indicates that βIII-tubulin functionally impacts glucose metabolism by suppressing glycolytic metabolism and reduces the reliance of these cells on glycolysis [161]. A study by Cicchillitti and colleagues has also pointed to a role for βIII-tubulin in glucose metabolism stress [138]. βIII-tubulin is upregulated in ovarian cancer cells treated with tunicamycin or wortmannin, to block protein glycosylation and PI3K signalling, respectively. Tunicamycin and wortmannin treatment also alters post-translational modifications of non-mitochondrial tubulins in cell lines with low basal βIII-tubulin expression [138]. In addition, ovarian cancer cells under glucose starvation have been shown to have an increased expression of βIII-tubulin and a decreased expression of βI-tubulin [130]. Upregulation of βIII-tubulin in these conditions correlates with HuR binding to the βIII-tubulin 3’UTR in ovarian cancer cells [130]. Moreover, βIII-tubulin expression promotes cell survival and proliferation in glucose-starved NSCLC cells [161]. Mechanistic detail of the role of this and other β-tubulin isotypes in regulating cancer cell metabolism remains to be fully defined [130].

### 4.4. Tubulin Isotypes and Mitochondrial Function

The microtubule network regulates the mitochondrial membrane potential and mitochondrial trafficking by as yet undefined mechanisms [reviewed in [162]]. Tubulin is an integral component of mitochondrial membranes [143,144,163], and these membranes are enriched with βIII-tubulin [138,144,164]. An important role for this isotype in mitochondrial dynamics and function is suggested from interactions between βIII-tubulin and GRP75 and mitochondrial-localised Hsp70 in Ovarian cancer cells [138]. Post-translational mechanisms are thought to regulate tubulin localization to mitochondria, with a non-phosphorylated and non-glycosylated form of βIII-tubulin present in mitochondria [138], however there was no correlation between high levels of mitochondrial βIII-tubulin localisation and tubulin binding agent resistance [138]. Further functional studies defining the importance of these protein–protein interactions and post-translational modifications are warranted.

Mitochondrial dynamics can significantly impact tumour progression [165,166] and growing evidence supports a role for the βII- and βIII-tubulin isotypes in regulating these complex processes. The βII-tubulin isotype is involved in mitochondrial localisation and metabolism in cardiomyocytes [167,168,169,170]. The βIII-tubulin C-terminal tail reduces kinesin-1 processivity and alters kinesin-1 force production in an isotype specific manner [171,172] and kinesin-1 is an important mediator of mitochondrial localisation [173,174]. Mutations within helix 12 of tubulin adjacent to the βIII-tubulin C-terminal tail also results in neurodevelopmental defects that are similar to those caused by kinesin mutations and abnormal mitochondrial trafficking [175,176,177,178]. Evaluation of the contribution of these two isotypes in mitochondrial dynamics may reveal functional implications for these proteins in disease progression and chemotherapy sensitivity [179].

### 4.5. Tubulin Isotypes and Endoplasmic Reticulum Stress

Tubulins are known to regulate endoplasmic reticulum (ER) homeostasis, with ER dynamics tightly linked to microtubule dynamics [180,181,182,183,184,185,186]. This is particularly important during ER stress, as expansion of the ER is one mechanism of relieving ER stress [187,188], however the involvement of specific tubulin isotypes in regulating ER stress is unclear. Suppression of βIII-tubulin expression promotes ER stress in response to glucose starvation, and in doing so promotes cell death and reduces cell proliferation [161]. βIII-Tubulin interacts with the master regulator of the ER stress response GRP78 in ovarian and NSCLC cancer cells [138,161] and influences the formation of a complex between AKT and GRP78 in response to glucose starvation [161], raising the possibility that it may play a role in maintaining the protein processing capacity of the ER to support continued cell growth in the harsh tumour microenvironment. Increasing recognition of the role of ER stress and the ER stress response in regulating chemotherapeutic efficacy and tumour progression [189,190,191] highlights that an involvement for tubulin isotypes in regulating these processes may enable drug resistance and promote tumour progression.

### 4.6. Tubulin Isotypes and Autophagy

The microtubule network is involved in the initiation and execution of macroautophagy (reviewed in [162]), which is an important regulator of cancer cell survival and their treatment sensitivity. The autophagy machinery operates in a highly concerted manner to sequester cytoplasmic components within an autophagosome and regulate the fusion of this autophagosome with lysosome to degrade its contents [192]. Recent studies have suggested a role for autophagy in regulating cancer cell survival and treatment sensitivity and reviews have covered this topic in detail [193,194,195]. Microtubules sequester autophagic components to regulate the initiation of autophagy [196,197,198,199,200], are involved in autophagosome trafficking [198] and in the fusion of autophagosomes with lysosomes to complete the pathway [201,202,203]. It has also been suggested that microtubules facilitate autophagosome formation. Dynamics of microtubules play a major role in the regulation of starvation-induced [198,202] autophagosome formation but not in basal conditions [202,204,205]. Although the involvement of particular tubulin isotypes in these processes is unclear, a recent study proposed βIII-tubulin as a binding partner with the key autophagic mediator LC3 [206]. The functional significance of this association, and indeed a role for other tubulin isotypes in autophagy, remain to be defined.

## 5. Tubulin and Metastasis

A growing body of evidence has pointed to a role for the tubulin proteins in the metastatic progression of cancer. Research conducted over the past decade has indicated several tubulin isotypes as potential prognostic markers whose expression levels in primary tumours are associated with aggressive disease with greater metastatic potential and the propensity of patients to suffer metastatic relapse, although the major focus has centred on βIII-tubulin expression. In particular, clinical data has identified that high βIII-tubulin protein expression strongly correlates with aggressive clinical behaviour and poor patient outcome in many forms of cancer, including breast, ovarian, gastric, glioblastoma, prostate, colorectal, pancreatic and NSCLC [39,47,66,121,132,207,208,209,210,211,212,213]. βIII-Tubulin expression is associated with high-grade malignancy in gliomas and gastric cancers [47,132,208,209,214,215]. Similarly, in NSCLC, expression of βIII-tubulin is associated with higher histological grades, poorly differentiated tumour tissue and progression to metastatic disease [39,207,211,212]. High βIII-tubulin expression is also detected at the invasive tumour edge of atypical carcinoid lung cancers [211], in lymph node metastases from primary adenocarcinoma NSCLC [216] and in metastatic lung cancers derived from colon, prostate and ovarian tumours but not from primary tumours of the breast [211]. In colorectal cancer, the expression of βIII-tubulin expression also correlates with high grade malignancy, tumour differentiation and lymphatic metastasis, suggesting a potential role of βIII-tubulin in prostate tumour differentiation and metastasis [213]. Overexpression of βIII-tubulin is observed in breast cancer brain metastases and its expression is significantly associated with distant metastases [121]. Furthermore, in prostate cancer, βIII-tubulin expression is associated with both *TMPRSS2:ERG* rearrangement, ERG expression and *PTEN* deletions, three key oncogenetic features in metastatic prostate cancer [48].

In melanoma, βIIb-tubulin mRNA transcript levels were significantly elevated in melanoma multinucleated giant cells which are frequently observed in metastatic sites and correlate with poor prognosis, indicating that this tubulin isotype may play an important role in melanoma metastasis [217]. Increased expression of βIVb-tubulin was observed in sentinel lymph node micrometastasis of colorectal cancer [218]. Similarly, in lung cancer cells, βIVa-tubulin expression is induced under non-adherent conditions, which resemble those experienced by circulating tumour cells [219].

Tumour metastasis requires completion of a multistep process, with dissemination of cancer cells into distant organ sites and their subsequent adaptation to foreign tissue microenvironments. Altered tubulin isotype composition may increase the metastatic potential of cancer cells by altering their capacity for migration and invasion. Emerging evidence indicates that activation of the epithelial-to-mesenchymal transition (EMT) program is critical in regulating invasion and metastasis [220] and that EMT reprogramming is influenced by the tubulin isotype composition. In particular, βIII-tubulin expression correlates with Snail expression levels and modulates the behaviour of Snail overexpression during EMT transition in colon cancer cells [221]. Knockdown of βIII-tubulin expression using RNA interference agents caused modulation of colon cancer cell movement and a decrease in their ability to migrate and invade [221]. Similarly, in breast cancer, a positive feedback regulation of ZEB1 and β-tubulin isotype classes I, III, and IVB has been reported [71]. Knockdown of ZEB1 in human breast cancer cells reduces β-tubulin isotype classes I, III, and IVB mRNA, whereas upregulation of ZEB1 was associated with increases in these isotype classes. This finding suggests that βIII-tubulin is a biomarker for cell survival mediated through ZEB1-induced EMT that contributes to the aggressive tumour phenotype in breast cancer.

Functional links between βIII-tubulin and metastasis have also been reported using lung and pancreatic cancer mouse models [63,66,123]. Knockdown of βIII-tubulin decreases anchorage independent growth, a major phenotype with respect to metastatic potential, in NSCLC cells [63]. βIII-tubulin suppression upregulates the adhesion-associated tumour suppressor Maspin, inhibits tumour spheroid outgrowth, cell migration, and sensitises NSCLC cells to anoikis [123]. Silencing βIII-tubulin expression also reduced pancreatic cancer cell growth, tumorigenic potential and metastatic burden [66]. Similarly, Kanojia and colleagues [121] demonstrated a role for βIII-tubulin in conferring brain metastatic potential to breast cancer cells by regulating several key signalling molecules involved in cell adhesion and metastasis [121]. Suppression of βIII-tubulin expression regulated the expression of L1CAM and β3-integrin to reduce extracellular matrix (ECM) attachment and signalling through the β3-integrin/(focal adhesion kinase) FAK/Src pathway in vitro. This was associated with reduced metastatic ability in vivo and improved survival in a brain metastasis model [121]. Overall, these findings imply a role of tubulin isotypes, in particular βIII-tubulin, as mediators of metastasis and may be potential predictive markers for neoplastic progression and patient outcome. However, the mechanisms by which βIII-tubulin regulate the metastatic cascade remain to be fully determined.

## 6. Conclusions

**Figure 1:**
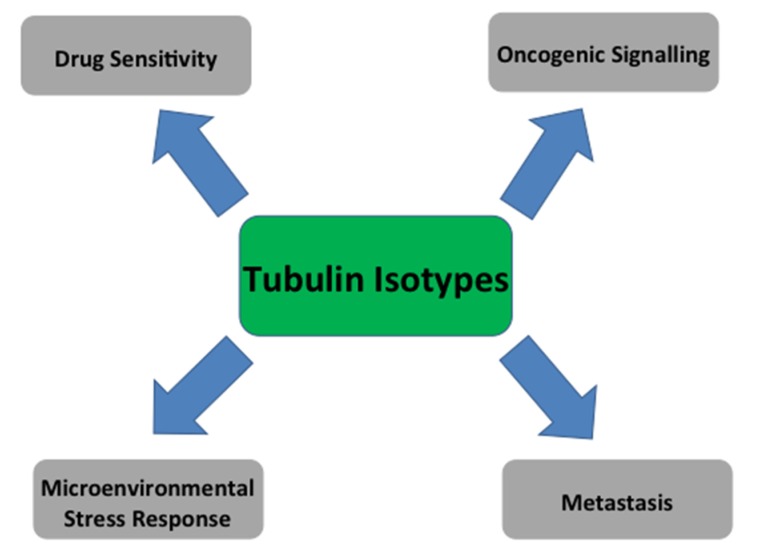
Tubulin isotypes influence multiple aspects of tumour biology and treatment sensitivity.

The aberrant expression of β-tubulin isotypes in a range of cancers is correlated with aggressive, drug refractory disease with poor clinical outcome. While the effect of isotype composition on the interaction of tubulin binding agents with their targets, and on microtubule dynamics may partially explain drug resistance to these classes of common chemotherapeutics, the current evidence suggests that tubulin isotypes are involved more broadly in biological processes that modulate tumour progression and metastasis. Through interactions with oncogenic signalling and cell survival programs that facilitate adaptation to the harsh tumour microenvironment, altered tubulin isotype composition in cancer may contribute to drug resistance and more aggressive disease (Figure 1). Defining the complex interplay of factors that regulate the spatiotemporal localisation and function of tubulin isotypes would significantly contribute to dissecting the mechanisms by which the tubulin isotype composition impacts tumour biology and treatment resistance in cancer.

In order to develop a more comprehensive understanding of the tubulin code in cancer, the context in which tubulin isotypes impact clinical outcome in this disease will need to be more robustly defined both within and between different types of cancers. Critical to this will be a firm understanding of the mechanisms that drive the individual and collective expression of tubulin isotypes in health and disease. The advent of gene-editing technologies provides great potential in the selective manipulation of isotype expression while retaining the endogenous regulatory framework. Defining the larger intracellular network in which the microtubule system operates will no doubt reveal the complex integration of this component of the cytoskeletal network with fundamental cellular processes and reveal novel therapeutic strategies for targeting altered tubulin isotype expression in cancer.

## Figures and Tables

**Table 1:** Clinical studies of tubulin isotypes in drug resistance.

Alteration of Tubulin Isotype	Effect	Tumour Type	Reference
High βI-tubulin	Poor response to docetaxel treatment	Breast cancer	[17]
Low βII-tubulin expression	Correlates with poor response to taxane treatment or advanced stage disease	Breast and ovarian cancer	[19,22]
High βIII-tubulin expression	Poor survival, poor outcome for surgical resection or TBA response; Correlates with subtype	Non-small cell lung cancer (NSCLC)	[24,25,37,38,39,40,41,42,43,44,45]
	Correlates with poor survival, poor response to platinum and taxane treatment, advanced stage or aggressive disease	Ovarian cancer	[19,20,21,28,29,30,31,32,33]
	Favourable response to taxane treatment	Ovarian (Clear cell adenocarcinoma)	[34]
	Poor response to taxane treatment	Breast cancer	[17,46]
	Poor response to taxane/platinum treatment	Uterine serous carcinoma	[18]
	Poor response to taxane treatment	Gastric cancer	[26,47]
	Advanced disease and early recurrence	Prostate cancer	[48]
High βIVa-tubulin expression	Poor response to taxol treatment	Ovarian cancer	[29]
High βV-tubulin expression	Favourable response to taxane treatment	NSCLC	[27]

## Abbreviations

TBA	Tubulin binding agents
MAP	Microtubule associated protein

## References

[1] Nogales E. (2000). Structural insights into microtubule function. Annu. Rev. Biochem..

[2] Bowne-Anderson H., Hibbel A., Howard J. (2015). Regulation of microtubule growth and catastrophe: Unifying theory and experiment. Trends Cell Biol..

[3] Downing K.H., Nogales E. (1998). Tubulin structure: Insights into microtubule properties and functions. Curr. Opin. Struct. Biol..

[4] Janke C. (2014). The tubulin code: Molecular components, readout mechanisms, and functions. J. Cell Biol..

[5] Valiron O., Caudron N., Job D. (2001). Microtubule dynamics. Cell. Mol. Life Sci..

[6] Akhmanova A., Steinmetz M.O. (2015). Control of microtubule organization and dynamics: Two ends in the limelight. Nat. Rev. Mol. Cell. Biol..

[7] Zich J., Hardwick K.G. (2010). Getting down to the phosphorylated “nuts and bolts” of spindle checkpoint signalling. Trends Biochem. Sci..

[8] Jordan M.A., Wilson L. (2004). Microtubules as a target for anticancer drugs. Nat. Rev. Cancer.

[9] Gigant B., Wang C.G., Ravelli R.B.G., Roussi F., Steinmetz M.O., Curmi P.A., Sobel A., Knossow M. (2005). Structural basis for the regulation of tubulin by vinblastine. Nature.

[10] Luduena R.F. (2013). A hypothesis on the origin and evolution of tubulin. Int. Rev. Cell Mol. Biol..

[11] Luduena R.F. (1993). Are tubulin isotypes functionally significant. Mol. Biol. Cell.

[12] Verdier-Pinard P., Pasquier E., Xiao H., Burd B., Villard C., Lafitte D., Miller L.M., Angeletti R.H., Horwitz S.B., Braguer D. (2009). Tubulin proteomics: Towards breaking the code. Anal. Biochem..

[13] Sullivan K.F., Cleveland D.W. (1986). Identification of conserved isotype-defining variable region sequences for 4 vertebrate β-tubulin polypeptide classes. Proc. Natl. Acad. Sci. USA.

[14] Lewis S.A., Tian G.L., Cowan N.J. (1997). The α- and β-tubulin folding pathways. Trends Cell Biol..

[15] Serna M., Carranza G., Martin-Benito J., Janowski R., Canals A., Coll M., Zabala J.C., Valpuesta J.M. (2015). The structure of the complex between α-tubulin, TBCE and TBCB reveals a tubulin dimer dissociation mechanism. J. Cell Sci..

[16] Vilmar A., Garcia-Foncillas J., Huarriz M., Santoni-Rugiu E., Sorensen J.B. (2012). RT-PCR versus immunohistochemistry for correlation and quantification of ERCC1, BRCA1, TUBB3 and RRM1 in NSCLC. Lung Cancer.

[17] Hasegawa S., Miyoshi Y., Egawa C., Ishitobi M., Taguchi T., Tamaki Y., Monden M., Noguchi S. (2003). Prediction of response to docetaxel by quantitative analysis of class I and III β-tubulin isotype mRNA expression in human breast cancers. Clin. Cancer Res..

[18] Roque D.M., Bellone S., English D.P., Buza N., Cocco E., Gasparrini S., Bortolomai I., Ratner E., Silasi D.A., Azodi M. (2013). Tubulin-β-III overexpression by uterine serous carcinomas is a marker for poor overall survival after platinum/taxane chemotherapy and sensitivity to epothilones. Cancer.

[19] Ohishi Y., Oda Y., Basaki Y., Kobayashi H., Wake N., Kuwano M., Tsuneyoshi M. (2007). Expression of β-tubulin isotypes in human primary ovarian carcinoma. Gynecol. Oncol..

[20] Izutsu N., Maesawa C., Shibazaki M., Oikawa H., Shoji T., Sugiyama T., Masuda T. (2008). Epigenetic modification is involved in aberrant expression of class III β-tubulin, TUBB3, in ovarian cancer cells. Int. J. Oncol..

[21] Du J., Li B., Fang Y., Liu Y., Wang Y., Li J., Zhou W., Wang X. (2015). Overexpression of class III β-tubulin, sox2, and nuclear survivin is predictive of taxane resistance in patients with stage III ovarian epithelial cancer. BMC Cancer.

[22] Bernard-Marty C., Treilleux I., Dumontet C., Cardoso F., Fellous A., Gancberg D., Bissery M.C., Paesmans M., Larsimont D., Piccart M.J. (2002). Microtubule-associated parameters as predictive markers of docetaxel activity in advanced breast cancer patients: Results of a pilot study. Clin. Breast Cancer.

[23] Seve P., Reiman T., Lai R., Hanson J., Santos C., Johnson L., Dabbagh L., Sawyer M., Dumontet C., Mackey J.R. (2007). Class III β-tubulin is a marker of paclitaxel resistance in carcinomas of unknown primary site. Cancer Chemother. Pharmacol..

[24] Levallet G., Bergot E., Antoine M., Creveuil C., Santos A.O., Beau-Faller M., de Fraipont F., Brambilla E., Levallet J., Morin F. (2012). High TUBB3 expression, an independent prognostic marker in patients with early non-small cell lung cancer treated by preoperative chemotherapy, is regulated by K-RAS signaling pathway. Mol. Cancer Ther..

[25] Seve P., Isaac S., Tredan O., Souquet P.J., Pacheco Y., Perol M., Lafanechere L., Penet A., Peiller E.L., Dumontet C. (2005). Expression of class IIIβ-tubulin is predictive of patient outcome in patients with non-small cell lung cancer receiving vinorelbine-based chemotherapy. Clin. Cancer Res..

[26] Hwang J.E., Hong J.Y., Kim K., Kim S.H., Choi W.Y., Kim M.J., Jung S.H., Shim H.J., Bae W.K., Hwang E.C. (2013). Class III β-tubulin is a predictive marker for taxane-based chemotherapy in recurrent and metastatic gastric cancer. BMC Cancer.

[27] Christoph D.C., Kasper S., Gauler T.C., Loesch C., Engelhard M., Theegarten D., Poettgen C., Hepp R., Peglow A., Loewendick H. (2012). βV-tubulin expression is associated with outcome following taxane-based chemotherapy in non-small cell lung cancer. Br. J. Cancer.

[28] Roque D.M., Buza N., Glasgow M., Bellone S., Bortolomai I., Gasparrini S., Cocco E., Ratner E., Silasi D.A., Azodi M. (2014). Class III β-tubulin overexpression within the tumor microenvironment is a prognostic biomarker for poor overall survival in ovarian cancer patients treated with neoadjuvant carboplatin/paclitaxel. Clin. Exp. Metastasis.

[29] Kavallaris M., Kuo D.Y.S., Burkhart C.A., Regl D.L., Norris M.D., Haber M., Horwitz S.B. (1997). Taxol-resistant epithelial ovarian tumors are associated with altered expression of specific β-tubulin isotypes. J. Clin. Investig..

[30] Mozzetti S., Ferlini C., Concolino P., Filippetti F., Raspaglio G., Prislei S., Gallo D., Martinelli E., Ranelletti F.O., Ferrandina G. (2005). Class III β-tubulin overexpression is a prominent mechanism of paclitaxel resistance in ovarian cancer patients. Clin. Cancer Res..

[31] Ferrandina G., Zannoni G.F., Martinelli E., Paglia A., Gallotta V., Mozzetti S., Scambia G., Ferlini C. (2006). Class III β-tubulin overexpression is a marker of poor clinical outcome in advanced ovarian cancer patients. Clin. Cancer Res..

[32] De Donato M., Mariani M., Petrella L., Martinelli E., Zannoni G.F., Vellone V., Ferrandina G., Shahabi S., Scambia G., Ferlini C. (2012). Class III β-tubulin and the cytoskeletal gateway for drug resistance in ovarian cancer. J. Cell. Physiol..

[33] Su D., Smith S.M., Preti M., Schwartz P., Rutherford T.J., Menato G., Danese S., Ma S.L., Yu H., Katsaros D. (2009). Stathmin and tubulin expression and survival of ovarian cancer patients receiving platinum treatment with and without paclitaxel. Cancer.

[34] Aoki D., Oda Y., Hattori S., Taguchi K., Ohishi Y., Basaki Y., Oie S., Suzuki N., Kono S., Tsuneyoshi M. (2009). Overexpression of class IIIβ-tubulin predicts good response to taxane-based chemotherapy in ovarian clear cell adenocarcinoma. Clin. Cancer Res..

[35] Sale S., Sung R., Shen P.D., Yu K., Wang Y., Duran G.E., Kim J.H., Fojo T., Oefner P.J., Sikic B.I. (2002). Conservation of the class I β-tubulin gene in human populations and lack of mutations in lung cancers and paclitaxel-resistant ovarian cancers. Mol. Cancer Ther..

[36] Wang W., Zhang H., Wang X., Patterson J., Winter P., Graham K., Ghosh S., Lee J.C., Katsetos C.D., Mackey J.R. (2016). Novel mutations involving βI-, βIIA-, or βIVB-tubulin isotypes with functional resemblance to βIII-tubulin in breast cancer. Protoplasma.

[37] Zhang H.L., Ruan L., Zheng L.M., Whyte D., Tzeng C.M., Zhou X.W. (2012). Association between class IIIβ-tubulin expression and response to paclitaxel/vinorebine-based chemotherapy for non-small cell lung cancer: A meta-analysis. Lung Cancer.

[38] Sève P., Lai R., Ding K., Winton T., Butts C., Mackey J., Dumontet C., Dabbagh L., Aviel-Ronen S., Seymour L. (2007). Class IIIβ-tubulin expression and benefit from adjuvant cisplatin/vinorelbine chemotherapy in operable non–small cell lung cancer: Analysis of NCIC JBR.10. Clin. Cancer Res..

[39] Reiman T., Lai R., Veillard A.S., Paris E., Soria J.C., Rosell R., Taron M., Graziano S., Kratzke R., Seymour L. (2012). Cross-validation study of class IIIβ-tubulin as a predictive marker for benefit from adjuvant chemotherapy in resected non-small-cell lung cancer: Analysis of four randomized trials. Ann. Oncol..

[40] Cucchiarelli V., Hiser L., Smith H., Frankfurter A., Spano A., Correia J.J., Lobert S. (2008). β-tubulin isotype classes II and V expression patterns in nonsmall cell lung carcinomas. Cell Motil. Cytoskelet..

[41] Azuma K., Sasada T., Kawahara A., Takamori S., Hattori S., Ikeda J., Itoh K., Yamada A., Kage M., Kuwano M. (2009). Expression of ERCC1 and ulin in non-small cell lung cancer patients treated with carboplatin and paclitaxel. Lung Cancer.

[42] Yang Y.L., Luo X.P., Xian L. (2014). The prognostic role of the class IIIβ-tubulin in non-small cell lung cancer (NSCLC) patients receiving the taxane/vinorebine-based chemotherapy: A meta-analysis. PLoS ONE.

[43] Guo N., Zhang W., Zhang B., Li Y., Tang J., Li S., Zhao Y., Zhao Y., Xia H., Yu C. (2015). EGFR and K-RAS mutations and ERCC1, TUBB3, TYMS, RRM1 and EGFR mRNA expression in non-small cell lung cancer: Correlation with clinical response to gefitinib or chemotherapy. Mol. Clin. Oncol..

[44] Ohashi T., Yoshimasu T., Oura S., Kokawa Y., Kawago M., Hirai Y., Miyasaka M., Aoishi Y., Kiyoi M., Nishiguchi H. (2015). Class IIIβ-tubulin expression in non-small cell lung cancer: A predictive factor for paclitaxel response. Anticancer Res..

[45] Jiang H., Yu X.M., Zhou X.M., Wang X.H., Su D. (2013). Correlation between microtubule-associated gene expression and chemosensitivity of patients with stage II non-small cell lung cancer. Exp. Ther. Med..

[46] Paradiso A., Mangia A., Chiriatti A., Tommasi S., Zito A., Latorre A., Schittulli F., Lorusso V. (2005). Biomarkers predictive for clinical efficacy of taxol-based chemotherapy in advanced breast cancer. Ann. Oncol..

[47] He W., Zhang D., Jiang J., Liu P., Wu C. (2014). The relationships between the chemosensitivity of human gastric cancer to paclitaxel and the expressions of class III β-tubulin, MAPT, and survivin. Med. Oncol..

[48] Tsourlakis M.C., Weigand P., Grupp K., Kluth M., Steurer S., Schlomm T., Graefen M., Huland H., Salomon G., Steuber T. (2014). βIII-tubulin overexpression is an independent predictor of prostate cancer progression tightly linked to ERG fusion status and PTEN deletion. Am. J. Pathol..

[49] Gan P.P., McCarroll J.A., Byrne F.L., Garner J., Kavallaris M. (2011). Specific β-tubulin isotypes can functionally enhance or diminish epothilone b sensitivity in non-small cell lung cancer cells. PLoS ONE.

[50] Gan P.P., Kavallaris M. (2008). Tubulin-targeted drug action: Functional significance of class II and class IVb β-tubulin in vinca alkaloid sensitivity. Cancer Res..

[51] Don S., Verrills N.M., Liaw T.Y.E., Liu M.L.M., Norris M.D., Haber M., Kavallaris M. (2004). Neuronal-associated microtubule proteins class IIIβ-tubulin and MAP2c in neuroblastoma: Role in resistance to microtubule-targeted drugs. Mol. Cancer Ther..

[52] Bhattacharya R., Cabral F. (2004). A ubiquitous β-tubulin disrupts microtubule assembly and inhibits cell proliferation. Mol. Biol. Cell.

[53] Lobert S., Jefferson B., Morris K. (2011). Regulation of β-tubulin isotypes by micro-RNA 100 in MCF7 breast cancer cells. Cytoskeleton.

[54] Verrills N.M., Walsh B.J., Cobon G.S., Hains P.G., Kavallaris M. (2003). Proteome analysis of vinca alkaloid response and resistance in acute lymphoblastic leukemia reveals novel cytoskeletal alterations. J. Biol. Chem..

[55] Mozzetti S., Iantomasi R., de Maria I., Prislei S., Mariani M., Camperchioli A., Bartollino S., Gallo D., Scambia G., Ferlini C. (2008). Molecular mechanisms of patupilone resistance. Cancer Res..

[56] Kanakkanthara A., Northcote P.T., Miller J.H. (2012). βII-tubulin and βIII-tubulin mediate sensitivity to peloruside A and laulimalide, but not paclitaxel or vinblastine, in human ovarian carcinoma cells. Mol. Cancer Ther..

[57] Wu H., Xie J., Pan Q., Wang B., Hu D., Hu X. (2013). Anticancer agent shikonin is an incompetent inducer of cancer drug resistance. PLoS ONE.

[58] Galmarini C.M., Kamath K., Vanier-Viornery A., Hervieu V., Peiller E., Falette N., Puisieux A., Jordan M.A., Dumontet C. (2003). Drug resistance associated with loss of p53 involves extensive alterations in microtubule composition and dynamics. Br. J. Cancer.

[59] Haber M., Burkhart C.A., Regl D.L., Madafiglio J., Norris M.D., Horwitz S.B. (1995). Altered expression of Mβ2, the class II β-tubulin isotype, in a murine J774.2 cell line with a high level of taxol resistance. J. Biol. Chem..

[60] Sharbeen G., McCarroll J., Liu J., Youkhana J., Limbri L.F., Biankin A.V., Johns A., Kavallaris M., Goldstein D., Phillips P.A. (2016). Delineating the role of βIV-tubulins in pancreatic cancer: βIVb-tubulin inhibition sensitizes pancreatic cancer cells to vinca alkaloids. Neoplasia.

[61] Narvi E., Jaakkola K., Winsel S., Oetken-Lindholm C., Halonen P., Kallio L., Kallio M.J. (2013). Altered TUBB3 expression contributes to the epothilone response of mitotic cells. Br. J. Cancer.

[62] Kavallaris M., Burkhart C.A., Horwitz S.B. (1999). Antisense oligonucleotides to class III β-tubulin sensitize drug-resistant cells to taxol. Br. J. Cancer.

[63] McCarroll J.A., Gan P.P., Liu M., Kavallaris M. (2010). β III-tubulin is a multifunctional protein involved in drug sensitivity and tumorigenesis in non-small cell lung cancer. Cancer Res..

[64] Mhaidat N.M., Thorne R.F., de Bock C.E., Zhang X.D., Hersey P. (2008). Melanoma cell sensitivity to docetaxel-induced apoptosis is determined by class III β-tubulin levels. FEBS Lett..

[65] Akasaka K., Maesawa C., Shibazaki M., Maeda F., Takahashi K., Akasaka T., Masuda T. (2009). Loss of class III β-tubulin induced by histone deacetylation is associated with chemosensitivity to paclitaxel in malignant melanoma cells. J. Investig. Dermatol..

[66] McCarroll J.A., Sharbeen G., Liu J., Youkhana J., Goldstein D., McCarthy N., Limbri L.F., Dischl D., Ceyhan G.O., Erkan M. (2015). β III-tubulin: A novel mediator of chemoresistance and metastases in pancreatic cancer. Oncotarget.

[67] Hari M., Yang H., Zeng C., Canizales M., Cabral F. (2003). Expression of class III β-tubulin reduces microtubule assembly and confers resistance to paclitaxel. Cell Motil. Cytoskelet..

[68] Blade K., Menick D.R., Cabral F. (1999). Overexpression of class I, II or IVb β-tubulin isotypes in CHO cells is insufficient to confer resistance to paclitaxel. J. Cell Sci..

[69] Ranganathan S., McCauley R.A., Dexter D.W., Hudes G.R. (2001). Modulation of endogenous β-tubulin isotype expression as a result of human β_III_cDNA transfection into prostate carcinoma cells. Br. J. Cancer.

[70] Gay D.A., Sisodia S.S., Cleveland D.W. (1989). Autoregulatory control of β-tubulin mRNA stability is linked to translation elongation. Proc. Natl. Acad. Sci. USA.

[71] Lobert S., Graichen M.E., Morris K. (2013). Coordinated regulation of β-tubulin isotypes and epithelial-to-mesenchymal transition protein zeb1 in breast cancer cells. Biochemistry.

[72] Seve P., Mackey J., Isaac S., Tredan O., Souquet P.J., Perol M., Lai R., Voloch A., Dumontet C. (2005). Class III β-tubulin expression in tumor cells predicts response and outcome in patients with non-small cell lung cancer receiving paclitaxel. Mol. Cancer Ther..

[73] Panda D., Miller H.P., Banerjee A., Luduena R.F., Wilson L. (1994). Microtubule dynamics in vitro are regulated by the tubulin isotype composition. Proc. Natl. Acad. Sci. USA.

[74] Derry W.B., Wilson L., Khan I.A., Luduena R.F., Jordan M.A. (1997). Taxol differentially modulates the dynamics of microtubules assembled from unfractionated and purified β-tubulin isotypes. Biochemistry.

[75] Falconer M.M., Echeverri C.J., Brown D.L. (1992). Differential sorting of β tubulin isotypes into colchicine-stable microtubules during neuronal and muscle differentiation of embryonal carcinoma-cells. Cell Motil. Cytoskelet..

[76] Rezania V., Azarenko O., Jordan M.A., Bolterauer H., Luduena R.F., Huzil J.T., Tuszynski J.A. (2008). Microtubule assembly of isotypically purified tubulin and its mixtures. Biophys. J..

[77] Freedman H., Huzil J.T., Luchko T., Luduena R.F., Tuszynski J.A. (2009). Identification and characterization of an intermediate taxol binding site within microtubule nanopores and a mechanism for tubulin isotype binding selectivity. J. Chem. Inf. Model..

[78] Pamula M.C., Ti S.C., Kapoor T.M. (2016). The structured core of human β tubulin confers isotype-specific polymerization properties. J. Cell Biol..

[79] Banerjee A., Roach M.C., Trcka P., Luduena R.F. (1990). Increased microtubule assembly in bovine brain tubulin lacking the type-III isotype of β-tubulin. J. Biol. Chem..

[80] Lu Q., Luduena R.F. (1993). Removal of β_III_ isotype enhances taxol-induced microtubule assembly. Cell Struct. Funct..

[81] Banerjee A., Luduena R.F. (1992). Kinetics of colchicine binding to purified β-tubulin isotypes from bovine brain. J. Biol. Chem..

[82] Gan P.P., McCarroll J.A., Po’uha S.T., Kamath K., Jordan M.A., Kavallaris M. (2010). Microtubule dynamics, mitotic arrest, and apoptosis: Drug-induced differential effects of β III-tubulin. Mol. Cancer Ther..

[83] Bouchet B.P., Puisieux A., Galmarini C.M. (2011). β III-tubulin is required for interphase microtubule dynamics in untransformed human mammary epithelial cells. Eur. J. Cell Biol..

[84] Khan I.A., Ludueña R.F. (2003). Different effects of vinblastine on the polymerization of isotypically purified tubulins from bovine brain. Investig. New Drugs.

[85] Wilson L., Lopus M., Miller H.P., Azarenko O., Riffle S., Smith J.A., Jordan M.A. (2015). Effects of eribulin on microtubule binding and dynamic instability are strengthened in the absence of the β III tubulin isotype. Biochemistry.

[86] Lopus M., Smiyun G., Miller H., Oroudjev E., Wilson L., Jordan M.A. (2015). Mechanism of action of ixabepilone and its interactions with the βIII-tubulin isotype. Cancer Chemother. Pharmacol..

[87] Kamath K., Wilson L., Cabral F., Jordan M.A. (2005). β III-tubulin induces paclitaxel resistance in association with reduced effects on microtubule dynamic instability. J. Biol. Chem..

[88] Stengel C., Newman S.P., Leese M.P., Potter B.V.L., Reed M.J., Purohit A. (2010). Class III β-tubulin expression and in vitro resistance to microtubule targeting agents. Br. J. Cancer.

[89] Banerjee A., Dhoore A., Engelborghs Y. (1994). Interaction of desacetamidocolchicine, a fast binding analog of colchicine with isotypically pure tubulin dimers αβII, αβIII and αβIV. J. Biol. Chem..

[90] Banerjee A., Engelborghs Y., Dhoore A., Fitzgerald T.J. (1997). Interactions of a bicyclic analog of colchicine with β-tubulin isoforms αβII, αβIII and αβIV. Eur. J. Biochem..

[91] Xu K., Schwarz P.M., Luduena R.F. (2002). Interaction of nocodazole with tubulin isotypes. Drug Dev. Res..

[92] Laing N., Dahllöf B., Hartley-Asp B., Ranganathan S., Tew K.D. (1997). Interaction of estramustine with tubulin isotypes. Biochemistry.

[93] Tulub A.A., Stefanov V.E. (2001). Cisplatin stops tubulin assembly into microtubules. A new insight into the mechanism of antitumor activity of platinum complexes. Int. J. Biol. Macromol..

[94] Boekelheide K., Arcila M.E., Eveleth J. (1992). CIS-diamminedichloroplatinum (II) (cisplatin) alters microtubule assembly dynamics. Toxicol. Appl. Pharmacol..

[95] Gan P.P., Pasquier E., Kavallaris M. (2007). Class III β-tubulin mediates sensitivity to chemotherapeutic drugs in non-small cell lung cancer. Cancer Res..

[96] Yeh L.-C.C., Banerjee A., Prasad V., Tuszynski J.A., Weis A.L., Bakos T., Yeh I.T., Ludueña R.F., Lee J.C. (2016). Effect of CH-35, a novel anti-tumor colchicine analogue, on breast cancer cells overexpressing the βIII isotype of tubulin. Investig. New Drugs.

[97] Kumbhar B.V., Borogaon A., Panda D., Kunwar A. (2016). Exploring the origin of differential binding affinities of human tubulin isotypes αβII, αβIII and αβIV for DAMA-colchicine using homology modelling, molecular docking and molecular dynamics simulations. PLoS ONE.

[98] Das L., Bhattacharya B., Basu G. (2012). Rationalization of paclitaxel insensitivity of yeast β-tubulin and human βIII-tubulin isotype using principal component analysis. BMC Res. Notes.

[99] Geyer E.A., Burns A., Lalonde B.A., Ye X., Piedra F.-A., Huffaker T.C., Rice L.M. (2015). A mutation uncouples the tubulin conformational and GTpase cycles, revealing allosteric control of microtubule dynamics. eLife.

[100] Ti S.C., Pamula M.C., Howes S.C., Duellberg C., Cade N.I., Kleiner R.E., Forth S., Surrey T., Nogales E., Kapoor T.M. (2016). Mutations in human tubulin proximal to the kinesin-binding site alter dynamic instability at microtubule plus- and minus-ends. Dev. Cell.

[101] Pal D., Mahapatra P., Manna T., Chakrabarti P., Bhattacharyya L., Banerjee A., Basu G., Roy S. (2001). Conformational properties of -tubulin tail peptide: Implications for tail-body interaction. Biochemistry.

[102] Freedman H., Luchko T., Luduena R.F., Tuszynski J.A. (2011). Molecular dynamics modeling of tubulin C-terminal tail interactions with the microtubule surface. Proteins Struct. Funct. Bioinf..

[103] Luchko T., Huzil J.T., Stepanova M., Tuszynski J. (2008). Conformational analysis of the carboxy-terminal tails of human β-tubulin isotypes. Biophys. J..

[104] Bhattacharyya B., Sackett D.L., Wolff J. (1985). Tubulin, hybrid dimers and tubulin S. J. Biol. Chem..

[105] Wolff J., Sackett D.L., Knipling L. (1996). Cation selective promotion of tubulin polymerization by alkali metal chlorides. Protein Sci..

[106] Mejillano M.R., Himes R.H. (1991). Assembly properties of tubulin after carboxyl group modification. J. Biol. Chem..

[107] Mejillano M.R., Tolo E.T., Williams R.C., Himes R.H. (1992). The conversion of tubulin carboxyl groups to amides has a stabilizing effect on microtubules. Biochemistry.

[108] Szasz J., Yaffe M.B., Elzinga M., Blank G.S., Sternlicht H. (1986). Microtubule assembly is dependent on a cluster of basic residues in α-tubulin. Biochemistry.

[109] Sherman G., Rosenberry T.L., Sternlicht H. (1983). Identification of lysine residues essential for microtubule assembly—Demonstration of enhanced reactivity during reductive methylation. J. Biol. Chem..

[110] Serrano L., Delatorre J., Maccioni R.B., Avila J. (1984). Involvement of the carboxyl-terminal domain of tubulin in the regulation of its assembly. Proc. Natl. Acad. Sci. USA.

[111] Job D., Pabion M., Margolis R.L. (1985). Generation of microtubule stability subclasses by microtubule-associated proteins—Implications for the microtubule dynamic instability model. J. Cell Biol..

[112] Stromberg E., Wallin M. (1994). Differences in the effect of Ca^2+^ on isolated microtubules from cod and cow brain. Cell Motil. Cytoskelet..

[113] Lefevre J., Chernov K.G., Joshi V., Delga S., Toma F., Pastre D., Curmi P.A., Savarin P. (2011). The C-terminus of tubulin, a versatile partner for cationic molecules binding of τ, polyamines, and calcium. J. Biol. Chem..

[114] Littauer U.Z., Giveon D., Thierauf M., Ginzburg I., Ponstingl H. (1986). Common and distinct tubulin binding-sites for microtubule-associated proteins. Proc. Natl. Acad. Sci. USA.

[115] Chau M.F., Radeke M.J., de Ines C., Barasoain I., Kohlstaedt L.A., Feinstein S.C. (1998). The microtubule-associated protein τ cross-links to two distinct sites on each α and β tubulin monomer via separate domains. Biochemistry.

[116] Laurin Y., Eyer J., Robert C.H., Prevost C., Sacquin-Mora S. (2017). Mobility and core-protein binding patterns of disordered C-terminal tails in β-tubulin isotypes. Biochemistry.

[117] Joe P.A., Banerjee A., Luduena R.F. (2009). Roles of β-tubulin residues Ala^428^ and Thr^429^ in microtubule formation in vivo. J. Biol. Chem..

[118] Wieczorek M., Bechstedt S., Chaaban S., Brouhard G.J. (2015). Microtubule-associated proteins control the kinetics of microtubule nucleation. Nat. Cell Biol..

[119] Chanez B., Goncalves A., Badache A., Verdier-Pinard P. (2015). Eribulin targets a ch-TOG-dependent directed migration of cancer cells. Oncotarget.

[120] Lee K.M., Cao D., Itami A., Pour P.M., Hruban R.H., Maitra A., Ouellette M.M. (2007). Class III β-tubulin, a marker of resistance to paclitaxel, is overexpressed in pancreatic ductal adenocarcinoma and intraepithelial neoplasia. Histopathology.

[121] Kanojia D., Morshed R.A., Zhang L.J., Miska J.M., Qiao J., Kim J.W., Pytel P., Balyasnikova I.V., Lesniak M.S., Ahmed A.U. (2015). β III-tubulin regulates breast cancer metastases to the brain. Mol. Cancer Ther..

[122] Xiao M., Tang Y., Chen W.W., Wang Y.L., Yang L., Li X., Song G.L., Kuang J. (2016). TUBB3 regulation by the ERK and AKT signaling pathways: A mechanism involved in the effect of arginine ADP-ribosyltransferase 1 (Art1) on apoptosis of colon carcinoma CT26 cells. Tumour Biol..

[123] McCarroll J.A., Gan P.P., Erlich R.B., Liu M., Dwarte T., Sagnella S.S., Akerfeldt M.C., Yang L., Parker A.L., Chang M.H. (2015). TUBB3/β III-tubulin acts through the PTEN/AKT signaling axis to promote tumorigenesis and anoikis resistance in non-small cell lung cancer. Cancer Res..

[124] Hu J.Y., Chu Z.G., Han J., Dang Y.M., Yan H., Zhang Q., Liang G.P., Huang Y.S. (2010). The p38/MAPK pathway regulates microtubule polymerization through phosphorylation of MAP4 and OP18 in hypoxic cells. Cell. Mol. Life Sci..

[125] Fang Y.D., Xu X., Dang Y.M., Zhang Y.M., Zhang J.P., Hu J.Y., Zhang Q., Dai X., Teng M., Zhang D.X. (2011). MAP4 mechanism that stabilizes mitochondrial permeability transition in hypoxia: Microtubule enhancement and dynlt1 interaction with VDAC1. PLoS ONE.

[126] Yoon S.O., Shin S., Mercurio A.M. (2005). Hypoxia stimulates carcinoma invasion by stabilizing microtubules and promoting the RAB11 trafficking of the α6β4 integrin. Cancer Res..

[127] Raspaglio G., Filippetti F., Prislei S., Penci R., de Maria I., Cicchillitti L., Mozzetti S., Scambia G., Ferlini C. (2008). Hypoxia induces class III β-tubulin gene expression by HIF-1α binding to its 3’ flanking region. Gene.

[128] Bordji K., Grandval A., Cuhna-Alves L., Lechapt-Zalcman E., Bernaudin M. (2014). Hypoxia-inducible factor-2 (HIF-2), but not HIF-1, is essential for hypoxic induction of class III β-tubulin expression in human glioblastoma cells. FEBS J..

[129] Raspaglio G., Petrillo M., Martinelli E., Puma D.D.L., Mariani M., de Donato M., Filippetti F., Mozzetti S., Prislei S., Zannoni G.F. (2014). Sox9 and HIF-2α regulate *TUBB3* gene expression and affect ovarian cancer aggressiveness. Gene.

[130] Raspaglio G., De Maria I., Filippetti F., Martinelli E., Zannoni G.F., Prislei S., Ferrandina G., Shahabi S., Scambia G., Ferlini C. (2010). Hur regulates β-tubulin isotype expression in ovarian cancer. Cancer Res..

[131] Hinman M.N., Lou H. (2008). Diverse molecular functions of Hu proteins. Cell. Mol. Life Sci..

[132] Katsetos C.D., del Valle L., Geddes J.F., Assimakopoulou M., Legido A., Boyd J.C., Balin B., Parikh N.A., Maraziotis T., de Chadarevian J.P. (2001). Aberrant localization of the neuronal class III β-tubulin in astrocytomas—A marker for anaplastic potential. Arch. Pathol. Lab. Med..

[133] Patel V.P., Chu C.T. (2014). Decreased SIRT2 activity leads to altered microtubule dynamics in oxidatively-stressed neuronal cells: Implications for Parkinson’s disease. Exp. Neurol..

[134] Drum B.M.L., Yuan C., Li L., Liu Q.H., Wordeman L., Santana L.F. (2016). Oxidative stress decreases microtubule growth and stability in ventricular myocytes. J. Mol. Cell. Cardiol..

[135] Joe P.A., Banerjee A., Luduena R.F. (2008). The roles of Cys124 and Ser239 in the functional properties of human βIII tubulin. Cell Motil. Cytoskelet..

[136] Kim J.S., Lee J.H., Jeong W.W., Choi D.H., Cha H.J., Kim D.H., Kwon J.K., Park S.E., Park J.H., Cho H.R. (2008). Reactive oxygen species-dependent endog release mediates cisplatin-induced caspase-independent apoptosis in human head and neck squamous carcinoma cells. Int. J. Cancer.

[137] Selimovic D., Hassan M., Haikel Y., Hengge U.R. (2008). Taxol-induced mitochondrial stress in melanoma cells is mediated by activation of c-jun n-terminal kinase (JNK) and p38 pathways via uncoupling protein 2. Cell. Signal..

[138] Cicchillitti L., Penci R., Di Michele M., Filippetti F., Rotilio D., Donati M.B., Scambia G., Ferlini C. (2008). Proteomic characterization of cytoskeletal and mitochondrial class III β-tubulin. Mol. Cancer Ther..

[139] Guo J.Y., Walss-Bass C., Luduena R.F. (2010). The β isotypes of tubulin in neuronal differentiation. Cytoskeleton.

[140] Infante A.S., Stein M.S., Zhai Y., Borisy G.G., Gundersen G.G. (2000). Detyrosinated (Glu) microtubules are stabilized by an ATP-sensitive plus-end cap. J. Cell Sci..

[141] Aon M.A., Cortassa S. (2002). Coherent and robust modulation of a metabolic network by cytoskeletal organization and dynamics. Biophys. Chem..

[142] Sheldon K.L., Maldonado E.N., Lemasters J.J., Rostovtseva T.K., Bezrukov S.M. (2011). Phosphorylation of voltage-dependent anion channel by serine/threonine kinases governs its interaction with tubulin. PLoS ONE.

[143] Rostovtseva T.K., Sheldon K.L., Hassanzadeh E., Monge C., Saks V., Bezrukov S.M., Sackett D.L. (2008). Tubulin binding blocks mitochondrial voltage-dependent anion channel and regulates respiration. Proc. Natl. Acad. Sci. USA.

[144] Carre M., Andre N., Carles G., Borghi H., Brichese L., Briand C., Braguer D. (2002). Tubulin is an inherent component of mitochondrial membranes that interacts with the voltage-dependent anion channel. J. Biol. Chem..

[145] Maldonado E.N., Sheldon K.L., DeHart D.N., Patnaik J., Manevich Y., Townsend D.M., Bezrukov S.M., Rostovtseva T.K., Lemasters J.J. (2013). Voltage-dependent anion channels modulate mitochondrial metabolism in cancer cells- regulation by free tubulin and erastin. J. Biol. Chem..

[146] Gurnev P.A., Rostovtseva T.K., Bezrukov S.M. (2011). Tubulin-blocked state of VDAC studied by polymer and ATP partitioning. FEBS Lett..

[147] Sheldon K.L., Gurnev P.A., Bezrukov S.M., Sackett D.L. (2015). Tubulin tail sequences and post-translational modifications regulate closure of mitochondrial voltage-dependent anion channel (VDAC). J. Biol. Chem..

[148] Tsujimoto Y., Shimizu S. (2007). Role of the mitochondrial membrane permeability transition in cell death. Apoptosis.

[149] Galluzzi L., Morselli E., Kepp O., Vitale I., Rigoni A., Vacchelli E., Michaud M., Zischka H., Castedo M., Kroemer G. (2010). Mitochondrial gateways to cancer. Mol. Asp. Med..

[150] Vertessy B.G., Bankfalvi D., Kovacs J., Low P., Lehotzky A., Ovadi J. (1999). Pyruvate kinase as a microtubule destabilizing factor in vitro. Biochem. Biophys. Res. Commun..

[151] Marmillot P., Keith T., Srivastava D.K., Knull H.R. (1994). Effect of tubulin on the activity of the muscle isoenzyme of lactate-dehydrogenase. Arch. Biochem. Biophys..

[152] Durrieu C., Berniervalentin F., Rousset B. (1987). Microtubules bind glyceraldehyde-3-phosphate dehydrogenase and modulate its enzyme-activity and quaternary structure. Arch. Biochem. Biophys..

[153] Muronetz V.I., Wang Z.X., Keith T.J., Knull H.R., Srivastava D.K. (1994). Binding constants and stoichiometries of glyceraldehyde-3-phosphate dehydrogenase-tubulin complexes. Arch. Biochem. Biophys..

[154] Tisdale E.J., Kelly C., Artalejo C.R. (2004). Glyceraldehyde-3-phosphate dehydrogenase interacts with Rab2 and plays an essential role in endoplasmic reticulum to Golgi transport exclusive of its glycolytic activity. J. Biol. Chem..

[155] Cueille N., Blanc C.T., Riederer I.M., Riederer B.M. (2007). Microtubule-associated protein 1B binds glyceraldehyde-3-phosphate dehydrogenase. J. Proteom. Res..

[156] Zala D., Hinckelmann M.V., Yu H., da Cunha M.M.L., Liot G., Cordelieres F.P., Marco S., Saudou F. (2013). Vesicular glycolysis provides on-board energy for fast axonal transport. Cell.

[157] Glaser P.E., Han X.L., Gross R.W. (2002). Tubulin is the endogenous inhibitor of the glyceraldehyde 3-phosphate dehydrogenase isoform that catalyzes membrane fusion: Implications for the coordinated regulation of glycolysis and membrane fusion. Proc. Natl. Acad. Sci. USA.

[158] Tisdale E.J., Azizi F., Artalejo C.R. (2009). Rab2 utilizes glyceraldehyde-3-phosphate dehydrogenase and protein kinase c iota to associate with microtubules and to recruit dynein. J. Biol. Chem..

[159] Orosz F., Santamaria B., Ovadi J., Aragon J.J. (1999). Phosphofructokinase from dictyostelium discoideum is a potent inhibitor of tubulin polymerization. Biochemistry.

[160] Kovacs J., Low P., Pacz A., Horvath I., Olah J., Ovadi J. (2003). Phosphoenolpyruvate-dependent tubulin-pyruvate kinase interaction at different organizational levels. J. Biol. Chem..

[161] Parker A.L., Turner N., McCarroll J.A., Kavallaris M. (2016). βIII-tubulin alters glucose metabolism and stress response signaling to promote cell survival and proliferation in glucose-starved non-small cell lung cancer cells. Carcinogenesis.

[162] Parker A., Mccarroll J., Kavallaris M. (2014). Microtubules and their role in cellular stress in cancer. Front. Oncol..

[163] Jung D., Filliol D., Miehe M., Rendon A. (1993). Interaction of brain mitochondria with microtubules reconstituted from brain tubulin and MAP2 or TAU. Cell Motil. Cytoskelet..

[164] Rovini A., Savry A., Braguer D., Carre M. (2011). Microtubule-targeted agents: When mitochondria become essential to chemotherapy. Biochim. Biophys. Acta.

[165] Strohecker A.M., Guo J.Y., Karsli-Uzunbas G., Price S.M., Chen G.J., Mathew R., McMahon M., White E. (2013). Autophagy sustains mitochondrial glutamine metabolism and growth of Braf^V600E^—Driven lung tumors. Cancer Discov..

[166] Karsli-Uzunbas G., Guo J.Y., Price S., Teng X., Laddha S.V., Khor S., Kalaany N.Y., Jacks T., Chan C.S., Rabinowitz J.D. (2014). Autophagy is required for glucose homeostasis and lung tumor maintenance. Cancer Discov..

[167] Gonzalez-Granillo M., Grichine A., Guzun R., Usson Y., Tepp K., Chekulayev V., Shevchuk I., Karu-Varikmaa M., Kuznetsov A.V., Grimm M. (2012). Studies of the role of tubulin β II isotype in regulation of mitochondrial respiration in intracellular energetic units in cardiac cells. J. Mol. Cell. Cardiol..

[168] Guzun R., Karu-Varikmaa M., Gonzalez-Granillo M., Kuznetsov A.V., Michel L., Cottet-Rousselle C., Saaremae M., Kaambre T., Metsis M., Grimm M. (2011). Mitochondria-cytoskeleton interaction: Distribution of β-tubulins in cardiomyocytes and HL-1 cells. Biochim. Biophys. Acta.

[169] Varikmaa M., Bagur R., Kaambre T., Grichine A., Timohhina N., Tepp K., Shevchuk I., Chekulayev V., Metsis M., Boucher F. (2014). Role of mitochondria-cytoskeleton interactions in respiration regulation and mitochondrial organization in striated muscles. Biochim. Biophys. Acta.

[170] Anmann T., Varikmaa M., Timohhina N., Tepp K., Shevchuk I., Chekulayev V., Saks V., Kaambre T. (2014). Formation of highly organized intracellular structure and energy metabolism in cardiac muscle cells during postnatal development of rat heart. Biochim. Biophys. Acta.

[171] Sirajuddin M., Rice L.M., Vale R.D. (2014). Regulation of microtubule motors by tubulin isotypes and post-translational modifications. Nat. Cell Biol..

[172] Feizabadi M.S. (2016). The contribution of the C-terminal tails of microtubules in altering the force production specifications of multiple kinesin-1. Cell Biochem. Biophys..

[173] Pilling A.D., Horiuchi D., Lively C.M., Saxton W.M. (2006). Kinesin-1 and dynein are the primary motors for fast transport of mitochondria in drosophila motor axons. Mol. Biol. Cell.

[174] Tanaka Y., Kanai Y., Okada Y., Nonaka S., Takeda S., Harada A., Hirokawa N. (1998). Targeted disruption of mouse conventional kinesin heavy chain, KIF5b, results in abnormal perinuclear clustering of mitochondria. Cell.

[175] Tischfield M.A., Baris H.N., Wu C., Rudolph G., van Maldergem L., He W., Chan W.M., Andrews C., Demer J.L., Robertson R.L. (2010). Human TUBB3 mutations perturb microtubule dynamics, kinesin interactions, and axon guidance. Cell.

[176] Tischfield M.A., Engle E.C. (2010). Distinct α- and β-tubulin isotypes are required for the positioning, differentiation and survival of neurons: New support for the ‘multi-tubulin’ hypothesis. Biosci. Rep..

[177] Niwa S., Takahashi H., Hirokawa N. (2013). β-Tubulin mutations that cause severe neuropathies disrupt axonal transport. EMBO J..

[178] Chew S., Balasubramanian R., Chan W.M., Kang P.B., Andrews C., Webb B.D., MacKinnon S.E., Oystreck D.T., Rankin J., Crawford T.O. (2013). A novel syndrome caused by the E410K amino acid substitution in the neuronal β-tubulin isotype 3. Brain.

[179] Al-Mehdi A.B., Pastukh V.M., Swiger B.M., Reed D.J., Patel M.R., Bardwell G.C., Pastukh V.V., Alexeyev M.F., Gillespie M.N. (2012). Perinuclear mitochondrial clustering creates an oxidant-rich nuclear domain required for hypoxia-induced transcription. Sci. Signal..

[180] Goyal U., Blackstone C. (2013). Untangling the web: Mechanisms underlying ER network formation. Biochim. Biophys. Acta.

[181] Terasaki M., Chen L.B., Fujiwara K. (1986). Microtubules and the endoplasmic-reticulum are highly interdependent structures. J. Cell Biol..

[182] Waterman-Storer C.M., Salmon E.D. (1997). Microtubule dynamics: Treadmilling comes around again. Curr. Biol..

[183] Friedman J.R., Webster B.M., Mastronarde D.N., Verhey K.J., Voeltz G.K. (2010). ER sliding dynamics and ER-mitochondrial contacts occur on acetylated microtubules. J. Cell Biol..

[184] Lee C., Chen L.B. (1988). Dynamic behavior of endoplasmic-reticulum in living cells. Cell.

[185] Chen G.A., Gharib T.G., Wang H., Huang C.C., Kuick R., Thomas D.G., Shedden K.A., Misek D.E., Taylor J.M.G., Giordano T.J. (2003). Protein profiles associated with survival in lung adenocarcinoma. Proc. Natl. Acad. Sci. USA.

[186] Waterman-Storer C.M., Salmon E.D. (1998). Endoplasmic reticulum membrane tubules are distributed by microtubules in living cells using three distinct mechanisms. Curr. Biol..

[187] Sriburi R., Jackowski S., Mori K., Brewer J.W. (2004). XBP1: A link between the unfolded protein response, lipid biosynthesis, and biogenesis of the endoplasmic reticulum. J. Cell Biol..

[188] Schuck S., Prinz W.A., Thorn K.S., Voss C., Walter P. (2009). Membrane expansion alleviates endoplasmic reticulum stress independently of the unfolded protein response. J. Cell Biol..

[189] Guo L., Chen R., Ma N., Xiao H.B., Chen Y., Chen F., Mei J., Ding F.B., Zhong H. (2013). Phosphorylation of eIF2α suppresses cisplatin-induced A549 cell apoptosis via p38 inhibition. Cancer Biother. Radiopharm..

[190] Sun Q., Hua J., Wang Q., Xu W., Zhang J.X., Zhang J., Kang J.H., Li M.Q. (2012). Expressions of GRP78 and Bax associate with differentiation, metastasis, and apoptosis in non-small cell lung cancer. Mol. Biol. Rep..

[191] Wang M., Kaufman R.J. (2014). The impact of the endoplasmic reticulum protein-folding environment on cancer development. Nat. Rev. Cancer.

[192] Mizushima N. (2007). Autophagy: Process and function. Genes Dev..

[193] Guo J.Y., Xia B., White E. (2013). Autophagy-mediated tumor promotion. Cell.

[194] Sui X., Chen R., Wang Z., Huang Z., Kong N., Zhang M., Han W., Lou F., Yang J., Zhang Q. (2013). Autophagy and chemotherapy resistance: A promising therapeutic target for cancer treatment. Cell Death Dis..

[195] Macintosh R.L., Ryan K.M. (2013). Autophagy in tumour cell death. Semin. Cancer Biol..

[196] Mochizuki Y., Ohashi R., Kawamura T., Iwanari H., Kodama T., Naito M., Hamakubo T. (2013). Phosphatidylinositol 3-phosphatase myotubularin-related protein 6 (MTMR6) is regulated by small GTPase Rab1B in the early secretory and autophagic pathways. J. Biol. Chem..

[197] Sancak Y., Bar-Peled L., Zoncu R., Markhard A.L., Nada S., Sabatini D.M. (2010). Ragulator-rag complex targets mTORC1 to the lysosomal surface and is necessary for its activation by amino acids. Cell.

[198] Geeraert C., Ratier A., Pfisterer S.G., Perdiz D., Cantaloube I., Rouault A., Pattingre S., Proikas-Cezanne T., Codogno P., Pous C. (2010). Starvation-induced hyperacetylation of tubulin is required for the stimulation of autophagy by nutrient deprivation. J. Biol. Chem..

[199] Di Bartolomeo S., Corazzari M., Nazio F., Oliverio S., Lisi G., Antonioli M., Pagliarini V., Matteoni S., Fuoco C., Giunta L. (2010). The dynamic interaction of AMBRA1 with the dynein motor complex regulates mammalian autophagy. J. Cell Biol..

[200] Luo S.Q., Garcia-Arencibia M., Zhao R., Puri C., Toh P.P.C., Sadiq O., Rubinsztein D.C. (2012). Bim inhibits autophagy by recruiting beclin 1 to microtubules. Mol. Cell.

[201] Kimura S., Noda T., Yoshimori T. (2008). Dynein-dependent movement of autophagosomes mediates efficient encounters with lysosomes. Cell Struct. Funct..

[202] Kochl R., Hu X.W., Chan E.Y.W., Tooze S.A. (2006). Microtubules facilitate autophagosome formation and fusion of autophagosomes with endosomes. Traffic.

[203] Amenta J.S., Sargus M.J., Baccino F.M. (1977). Effect of microtubular or translational inhibitors on general cell protein degradation—Evidence for a dual catabolic pathway. Biochem. J..

[204] Aplin A., Jasionowski T., Tuttle D.L., Lenk S.E., Dunn W.A. (1992). Cytoskeletal elements are required for the formation and maturation of autophagic vacuoles. J. Cell. Physiol..

[205] Reunanen H., Marttinen M., Hirsimaki P. (1988). Effects of griseofulvin and nocodazole on the accumulation of autophagic vacuoles in ehrlich ascites tumor-cells. Exp. Mol. Pathol..

[206] Kraft L.J., Manral P., Dowler J., Kenworthy A.K. (2016). Nuclear LC3 associates with slowly diffusing complexes that survey the nucleolus. Traffic.

[207] Vilmar A.C., Santoni-Rugiu E., Sorensen J.B. (2011). Class III β-tubulin in advanced NSCLC of adenocarcinoma subtype predicts superior outcome in a randomized trial. Clin. Cancer Res..

[208] Katsetos C.D., Del Valle L., Geddes J.F., Aldape K., Boyd J.C., Legido A., Khalili K., Perentes E., Mork S.J. (2002). Localization of the neuronal class III β-tubulin in oligodendrogliomas: Comparison with Ki-67 proliferative index and 1p/19q status. J. Neuropathol. Exp. Neurol..

[209] Katsetos C.D., Draberova E., Smejkalova B., Reddy G., Bertrand L., de Chadarevian J.P., Legido A., Nissanov J., Baas P.W., Draber P. (2007). Class III β-tubulin and gamma-tubulin are co-expressed and form complexes in human glioblastoma cells. Neurochem. Res..

[210] Egevad L., Valdman A., Wiklund N.P., Seve P., Dumontet C. (2010). β-tubulin III expression in prostate cancer. Scand. J. Urol. Nephrol..

[211] Katsetos C.D., Kontogeorgos G., Geddes J.F., Herman M.M., Tsimara-Papastamatiou H., Yu Y.X., Sakkas L.I., Tsokos M., Patchefsky A.S., Ehya H. (2000). Differential distribution of the neuron-associated class III β-tubulin in neuroendocrine lung tumors. Arch. Pathol. Lab. Med..

[212] Seve P., Dumontet C. (2008). Is class III β-tubulin a predictive factor in patients receiving tubulin-binding agents?. Lancet Oncol..

[213] Zhao X., Yue C., Chen J., Tian C., Yang D., Xing L., Liu H., Jin Y. (2016). Class III β-tubulin in colorectal cancer: Tissue distribution and clinical analysis of chinese patients. Med. Sci. Monit..

[214] Shahabi S., He S., Kopf M., Mariani M., Petrini J., Scambia G., Ferlini C. (2013). Free testosterone drives cancer aggressiveness: Evidence from us population studies. PLoS ONE.

[215] Orsted D.D., Nordestgaard B.G., Bojesen S.E. (2014). Plasma testosterone in the general population, cancer prognosis and cancer risk: A prospective cohort study. Ann. Oncol..

[216] Katsetos C.D., Herman M.M., Mork S.J. (2003). Class III β-tubulin in human development and cancer. Cell Motil. Cytoskelet..

[217] Mi R., Pan C., Zhou Y., Liu Y., Jin G., Liu F. (2016). Identification of the metastasis potential and its associated genes in melanoma multinucleated giant cells using the PHA-ECM830 fusion method. Oncol. Rep..

[218] He Z.Y., Wen H., Shi C.B., Wang J. (2010). Up-regulation of hnRNP A1, Ezrin, tubulin β-2C and Annexin A1 in sentinel lymph nodes of colorectal cancer. World J. Gastroenterol..

[219] Atjanasuppat K., Lirdprapamongkol K., Jantaree P., Svasti J. (2015). Non-adherent culture induces paclitaxel resistance in H460 lung cancer cells via ERK-mediated up-regulation of βIVa-tubulin. Biochem. Biophys. Res. Commun..

[220] Thiery J.P., Acloque H., Huang R.Y., Nieto M.A. (2009). Epithelial-mesenchymal transitions in development and disease. Cell.

[221] Sobierajska K., Wieczorek K., Ciszewski W.M., Sacewicz-Hofman I., Wawro M.E., Wiktorska M., Boncela J., Papiewska-Pajak I., Kwasniak P., Wyroba E. (2016). β-III tubulin modulates the behavior of snail overexpressed during the epithelial-to-mesenchymal transition in colon cancer cells. Biochim. Biophys. Acta.

